# Merging of the Fukushima Health Management Survey With the National and Local Cancer Registry to Refine the Detection of Thyroid Cancer Cases After the 2011 Fukushima Daiichi Nuclear Power Plant Accident

**DOI:** 10.1002/cam4.70610

**Published:** 2025-02-06

**Authors:** Reiko Kimura‐Tsuchiya, Masanori Nagao, Shigehira Saji, Fumikazu Hayashi, Tetsuya Ohira, Hiroki Shimura, Fumihiko Furuya, Satoru Suzuki, Satoshi Suzuki, Tetsuo Ishikawa, Susumu Yokoya, Hitoshi Ohto, Seiji Yasumura

**Affiliations:** ^1^ Department of Medical Oncology Fukushima Medical University School of Medicine Fukushima Japan; ^2^ Radiation Medical Science Center for the Fukushima Health Management Survey Fukushima Medical University Fukushima Japan; ^3^ Department of Epidemiology Fukushima Medical University School of Medicine Fukushima Japan; ^4^ Department of Laboratory Medicine Fukushima Medical University School of Medicine Fukushima Japan; ^5^ Department of Thyroid and Endocrinology Fukushima Medical University School of Medicine Fukushima Japan; ^6^ Department of Radiation Physics and Chemistry Fukushima Medical University School of Medicine Fukushima Japan

**Keywords:** cancer registry, Fukushima health management survey, nuclear accident, thyroid cancer

## Abstract

**Background:**

After the Fukushima Daiichi Nuclear Power Plant accident in 2011, the Fukushima Health Management Survey (FHMS) was implemented in Fukushima Prefecture to promote long‐term health care. The FHMS included thyroid ultrasound examination (TUE) for individuals aged ≤ 18 years, including fetuses at the time of the accident. However, the FHMS may not have captured all cases of thyroid cancer because it only followed up with examinees. To address this gap, we aimed to merge individual‐level information from the FHMS with national and local cancer registries (CRs) to determine the limitations of the FHMS and CRs in capturing thyroid cancer cases.

**Methods:**

The FHMS‐eligible residents' information was supplemented by merging and cross‐validating the FHMS and CR data using the Fukushima Prefectural Cancer Registry (FPCR), 2008–2015, and the National Cancer Registry (NCR), 2016–2018. For analysis, registered cases were classified into three groups: registered in both the CR and FHMS, or only in the CRs, or only in the FHMS. The characteristics of each case were evaluated in each database.

**Results:**

In the FHMS, 212 thyroid cancer cases were identified through 2018, with another 42 cases identified in the CRs. Of the 176 thyroid cancer cases registered until 2015, 28 (15.9%) were identified in the FHMS only and 13 (7.4%) in the FPCR only. Of the 78 additional cases identified since 2016, 29 (37.2%) were identified in the NCR only and 6 (7.7%) in the FHMS only. This indicates that the NCR captured the cases more efficiently than the FPCR.

**Conclusion:**

Merging data from the FHMS and CRs at the individual level is necessary to capture thyroid cancer cases more accurately after the 2011 nuclear accident.

## 1 Introduction

The Great East Japan Earthquake of March 11, 2011, and the subsequent tsunami led to a radiation accident at the Fukushima Daiichi Nuclear Power Plant, requiring evacuation of the area due to health concerns. Considering historical data of an increased incidence of thyroid cancer in children and adolescents following the Chernobyl Nuclear Power Plant accident in 1986 [1, 2], there was a concern for a similar increase in pediatric cases of thyroid cancer in Fukushima. Although the radiation exposure levels in Fukushima were much lower than those in Chernobyl, the long‐term effects of low‐dose radiation exposure on the incidence of thyroid cancer have not been elucidated [3, 4, 5, 6, 7].

To promote the long‐term health of the residents of Fukushima Prefecture, the Fukushima Health Management Survey (FHMS) was implemented in Fukushima Prefecture 3 months after the nuclear accident. The FHMS evaluates individuals’ radiation dose exposure and collects general health information, with thyroid ultrasound examination (TUE) performed for individuals aged ≤ 18 years. Despite the relatively high participation rate in the FHMS, thyroid cancer cases not included in the FHMS have been diagnosed at Fukushima Medical University Hospital or other hospitals, with these cases labeled as “outside the FHMS cases.” [8] Although the FHMS program covers individuals living outside Fukushima Prefecture, it may be difficult to accurately detect thyroid cancer patients because more than 20,000 people have evacuated outside the prefecture. In addition to the FHMS, the Fukushima Prefecture Cancer Registry (FPCR) was launched in 2010 and was incorporated into the Japanese National Cancer Registry (NCR) in 2016. These two registries could be used to identify cases of thyroid cancer “outside of the FHMS.” Accordingly, our aim in this study was to merge individual‐level information from the FHMS with information from the cancer registries (CRs), which is a combination of the FPCR and NCR, and to determine differences in the number of thyroid cancer cases identified between the individual registries and the merged dataset. From this analysis, we investigated the limitations of the FHMS and the CRs in capturing cases of thyroid cancer after the nuclear accident.

## 2 Methods

### 2.1 Ethics Statement

This study was approved by the Ethics Review Committee of Fukushima Medical University (approval number: 1318, 2020–011) and conducted in accordance with the Helsinki Declaration of the World Medical Association. In the FHMS, written informed consent was obtained from the parents or guardians of all survey participants < 18 years of age and from the individuals themselves for those aged ≥ 18 years. Access to the NCR and FPCR datasets was obtained with permission from the governments of Japan and Fukushima Prefecture under the Cancer Registry Promotion Act. Data presented in all figures and tables in this study were independently processed by the authors. Based on the regulations for the use of cancer registry information, only proportions are reported when the number of cases is less than 3.

### 2.2 Registries Used for Thyroid Cancer

The FHMS was established to monitor the long‐term health outcomes of the 2011 radiation accident in the Fukushima Prefecture and includes the following components [9, 10, 11]: a TUE for individuals aged ≤ 18 years; a comprehensive health check; a mental health and lifestyle survey; and a pregnancy and birth survey. In the first round of examinations, TUE was performed in children and adolescents aged 0–18 years on April 1, 2011 (*n* = 367,637), from October 2011 to March 2014 [12, 13]. The second round of examinations included children born between April 2, 2011, and April 1, 2012 (*n* = 13,660), with TUEs conducted from April 2014 to March 2016 [14, 15]. A third round of TUEs was conducted from April 2016 to March 2017 [16]. For children and adolescents enrolled to undergo TUEs, examinations are repeated every 2 years until the age of 20 and every 5 years thereafter. The name and address of each individual are re‐registered at each TUE examination. The participation rates in the first, second, and third rounds of TUEs were 81.7%, 71.0%, and 64.7%, respectively [17]. Although the TUE participation rate has decreased in each round, the present study included all TUE‐eligible individuals aged < 18 years who lived in the Fukushima Prefecture in 2011 (including those who had never undergone TUE).

The NCR was established in 2016 in response to the 2013 Promotion of Cancer Registries Act (No. 111), with Article 21 allowing cancer researchers and health planning officers at national, prefectural, and local governments to access the registry data. Under this law, all hospitals and designated clinics must report information on cancer cases to the CR offices of each prefecture. Cancer cases identified in each prefecture are matched against data in the NCR to provide population‐based cancer incidence data and survival rates in Japan.

The FPCR was established under the Health Promotion Act (No. 103) of 2002 and the Cancer Control Act (No. 98) of 2006. The FPCR was implemented in 2010, with 17 participating hospitals, and included data starting from 2008; 97 hospitals participated in the FPCR in 2015. Of note, the FPCR only registers patients with thyroid cancer who live in the Fukushima Prefecture. Therefore, the FPCR does not capture most patients diagnosed with thyroid cancer who reside outside the Fukushima Prefecture, even if these individuals lived in the Fukushima Prefecture at the time of the 2011 radiation accident. The FPCR was included in the NCR in 2016.

### 2.3 Data Analysis

Individual information (name, address, and date of birth) for TUE‐eligible individuals, including those who have not previously participated in the TUE in the FHMS database was used to match cases in the NCR and FPCR databases. Of note, fetuses were included based on mothers’ certification of residence in Fukushima on March 11, 2011. The FPCR includes data from 2012 to 2015 and the NCR from 2016 to 2018.

**FIGURE 1:**
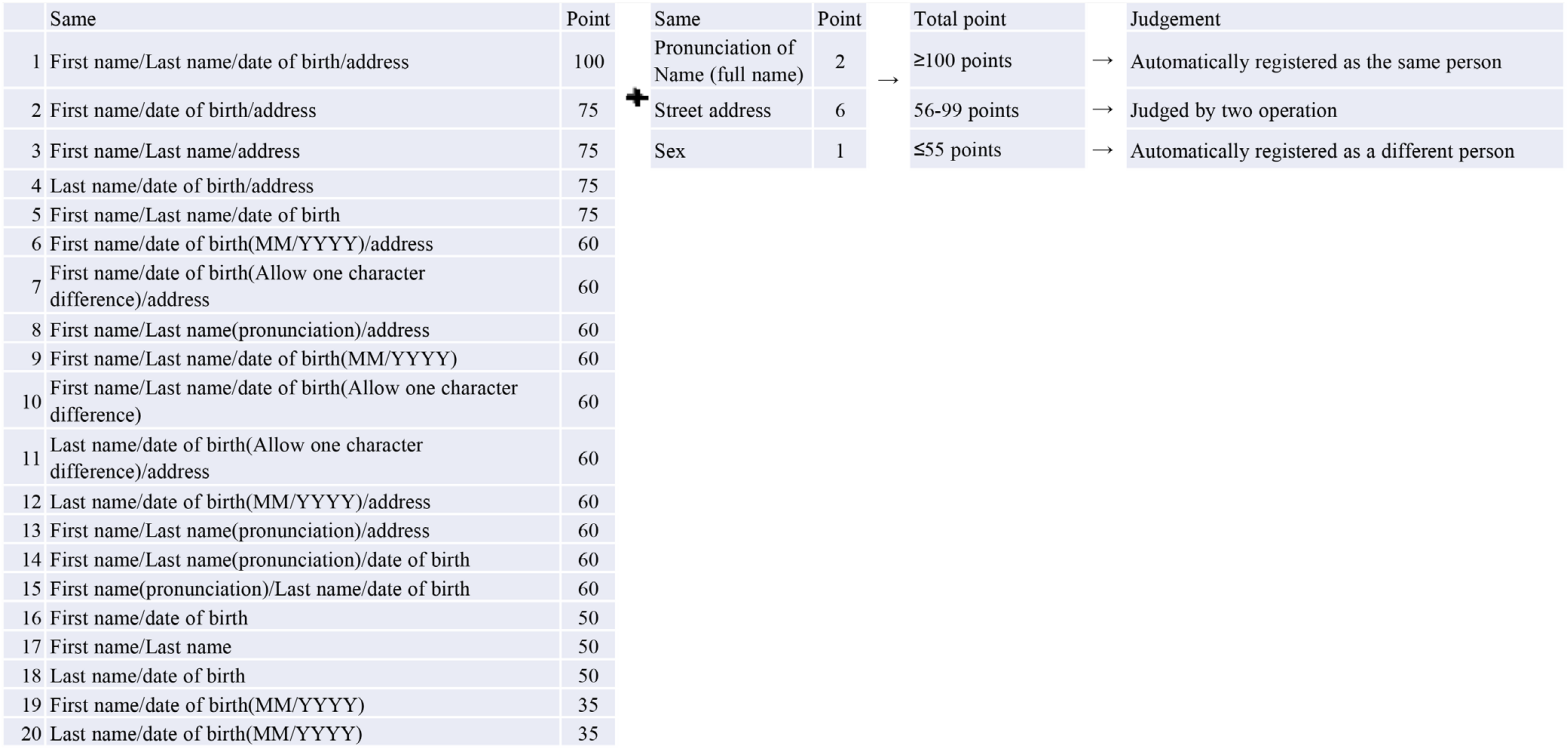
Criteria used for verification of cases between the Fukushima Health Management Survey (FHMS) and the National Cancer Registry (NCR) or Fukushima Prefecture Cancer Registry (FPCR). * If you select a point that matches the address in the right column (specifically, 1–4,6‐8,11–13), this addition will not be made.

Based on historical changes in last name and address for each of the approximately 380,000 TUE‐eligible cases, master records were created for merging with the CRs. This was done by multiplying all combinations of all names and addresses for each TUE‐eligible case. The list, amplified to 780,000, was submitted to the NCR and FCRP offices. The 780,000 master records were merged with CR records, using the criteria outlined in Figure 1, ensuring that no cases were missed. The researcher and the cancer registrar performed the merging and cross‐validation of cases. The 56–99 point cases, as described in Figure 1, were merged by referring to the 2017 and 2021 residential information. Of note, in Japan, a middle name is not routinely used, and women usually adopt their husband's family name after marriage, which limits the accuracy of the verification process.

**FIGURE 2:**
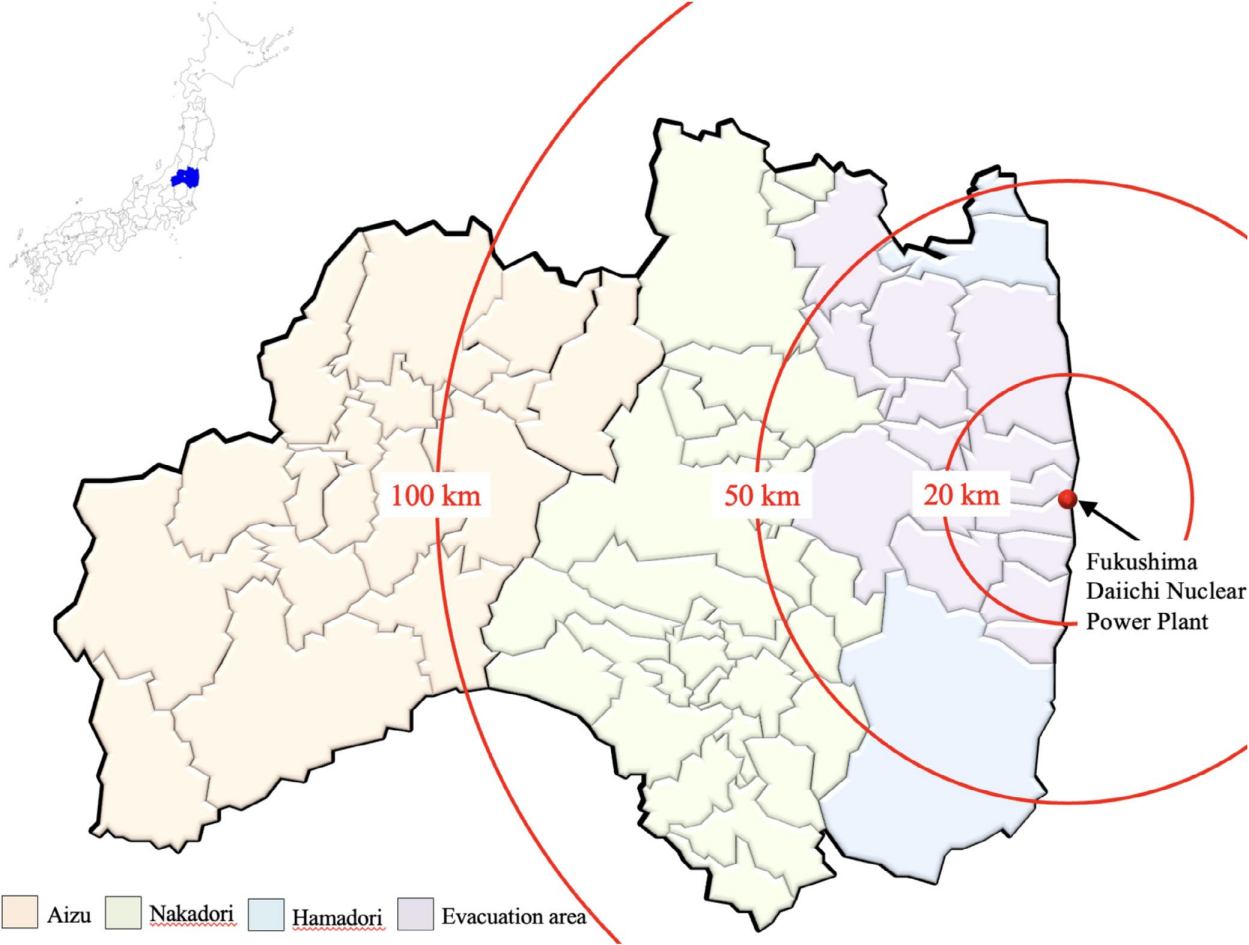
Study area, Fukushima Prefecture, Japan.

The anonymized data provided were classified as follows: cases registered in both the FHMS and CR (both group); cases registered in the FHMS but not the CR (only in the FHMS group); and cases registered in the CR but not the FHMS (only in the CR group). The characteristics of the three groups were compared, including the year of registration, age, sex, residential area at the time of the disaster, circumstances of cancer detection, and clinical stage of cancer, with this information being included in the CR. The classification of the residential area at the time of the disaster is shown in Figure 2. In the CR, the circumstances of cancer detection were selected from the following: cancer screening/health checkups/physical examination, accidental cancer detection during follow‐up of other diseases, autopsy, other, or unknown.

In the present study, we examined the reasons why cases were registered only in certain registries based on the factors mentioned above. To confirm the consistency in the number of thyroid cancer cases, the cumulative detection rate of thyroid cancer according to age at the time of the accident was calculated.

## 3 Results

Between 2012 and 2018, 212 cases of thyroid cancer were registered in the FHMS, with the number of cases increasing to 254 after the merging of the CRs datasets. Therefore, 42 cases of thyroid cancer were newly identified after merging the datasets. Following a review of the 42 cases registered only in the CR, 24 (57.1%) were identified as cancer detection during follow‐up for another health condition (Table 1). Among the 24 cases where thyroid cancer was incidentally detected during follow‐up for another health condition, 22 cases had previously undergone FHMS examination. In addition, 21 of 22 patients were found to have cysts ≥ 20.1 mm ornodules ≥ 5.1 mm as a result of TUE, and a detailed examination was recommended. There were no significant differences in sex and age at the time of the accident, or cancer clinical stage among the three groups.

**TABLE 1:** Characteristics of cases categorized into three groups.

	Both *n* = 178	Only in the FHMS *n* = 34	Only in the CR *n* = 42	*p*
Sex				0.373
Female *N* (%)	108 (60.7)	23 (67.6)	30 (71.4)	
Male *N* (%)	70 (39.3)	11 (32.4)	12 (28.6)	
Age at the time of the accident (years)				0.139
Median (Q1–Q3)	14 (11–16)	15 (13–17)	14 (11–16)	
Mean (SD)	13.4 (3.3)	14.6 (3.0)	13.3 (3.7)	
Age at the time of detection/registration (years)				0.033
Median (Q1–Q3)	18 (15–19)	19 (17–20)	18.5 (16–21)	
Mean (SD)	17.3 (3.0)	18.0 (2.8)	18.7 (3.8)	
Circumstances of cancer detection *N* (%)				< 0.001
Screening^a^	(> 95%)	—	12 (28.6)	
By chance^b^	4 (2.2)	—	24 (57.1)	
Other/unknown	(< 3%)	—	6 (14.3)	
Stage *N* (%)				0.287
Localized	63 (35.4)	—	19 (45.2)	
Other/unknown	115 (64.6)^c^	—	23 (54.8)^d^	

*Note:* Based on the regulations for the use of cancer registry information, only proportions are reported when the number of cases is fewer than three. Group comparisons were evaluated using Fisher's exact test for categorical variables and the Kruskal–Wallis test for continuous variables.

Abbreviations: CR, cancer registry; FHMS, Fukushima Health Management Survey.

^a^ Cancer screening, health checkups, or physical examinations.

^b^ Found incidentally during follow‐up for another health condition.

^c^ ≥ 95% of the 115 thyroid cancer cases detected had regional lymph node metastases.

^d^ ≥ 75% of the 23 thyroid cancer cases detected had regional lymph node metastases.

**FIGURE 3:**
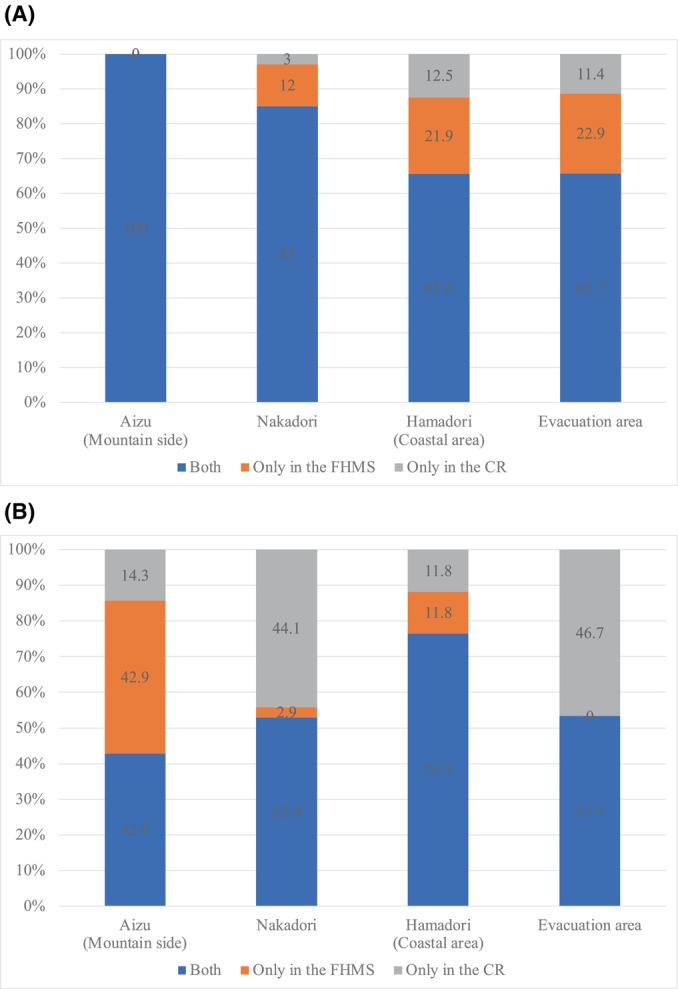
Addresses of cases registered in the Cancer Registry (CR) or Fukushima Health Management Survey (FHMS) at the time of the accident. *These figures include fewer than three cases, and only percentages are shown, following the regulations for the use of cancer registry information. (A) Cases registered between 2012 and 2015. The CR was the Fukushima Prefecture Cancer Registry. (B) Cases registered between 2016 and 2018. The CR was the National Cancer Registry.

The year of registration and region were analyzed to identify the reason for not capturing all thyroid cancer cases in the CRs. Until 2015, there was a greater number of cases in the FHMS group only, with the number of cases increasing in the CR group only after 2016 (Table 2). An examination by area of residence at the time of the accident found that about 20% of the cases were registered only in the FHMS in the Hamadori (coastal side) and evacuation area (Tamura City, Minamisoma City, Date City, Kawamata Town, Hirono Town, Naraha Town, Tomioka Town, Okuma Town, Futaba Town, Namie Town, Kawauchi Village, Katsurao Village, Iitate Village) until 2015 (Figure 3A). After 2016, the cases decreased in Hamadori and disappeared in the evacuated area (Figure 3B). On the other hand, Aizu showed a different pattern from the other regions, but the number of cases was too small to evaluate this tendency. Registration of cases tended to occur later in the CR compared to that in the FHMS (Table 3).

**TABLE 2:** Thyroid cancer cases by year of registration.

	All	Only in the FHMS	Only in the CR	Both
2012–2015 N (%)	176 (100.0)	28 (15.9)	13 (7.4)	135 (76.7)
2016–2018 N (%)	78 (100.0)	6 (7.7)	29 (37.2)	43 (55.1)
All *N* (%)	254 (100.0)	34 (13.4)	42 (16.5)	178 (70.1)

*Note:* 2012–2015: The Fukushima Prefecture Cancer Registry. 2016–2018: The National Cancer Registry.

Abbreviations: CR, cancer registry; FHMS, Fukushima Health Management Survey.

**TABLE 3:** Differences in the registration of thyroid cancer cases between the FHMS and the CR.

		Year of registration (CR)							
		2012	2013	2014	2015	2016	2017	2018	
Year of detection (FHMS)	2012	100							
	2013		94	6					
	2014			92	8				
	2015				82	18			(%)
	2016					77	23		
	2017						100		
	2018							100	

*Note:* The data show that 77% of the cases detected in the FHMS in 2016 were registered in the CR in 2016 and 23% in 2017.

* For patients whose cancer registration diagnosis date coincided with that of the secondary examination in the FHMS, the year of the FHMS diagnosis was used as the date of the secondary examination.

Abbreviations: CR, cancer registry, FHMS, Fukushima Health Management Survey.

**FIGURE 4:**
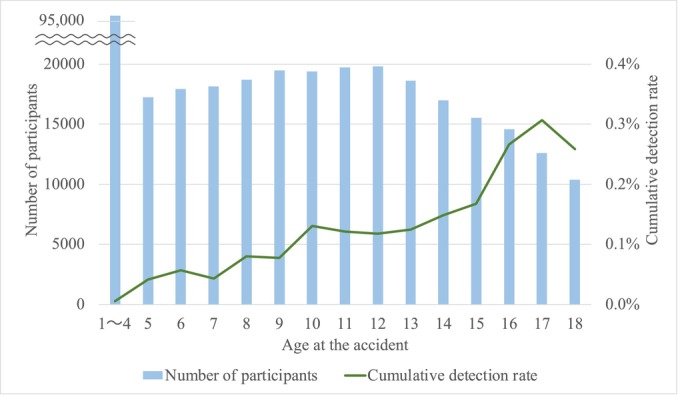
Cumulative detection rate according to age at the time of the accident.

Lastly, the cumulative detection rate is shown in Figure 4. Consistent with the age‐specific estimated incidence rate for thyroid cancer, there was a greater detection of thyroid cancer in older individuals than in younger individuals.

## 4 Discussion

Merging the FHMS and CRs led to the identification of 42 additional cases of newly detected thyroid cancer. Overall, the number of thyroid cancer cases identified was consistent with the prevalence of cases predicted by Takahashi et al. [18] using a cancer progression model with simulations based on outcomes of the first round of TUEs in the FHMS.

The older age of the FHMS‐only registrants may be due to many people leaving Fukushima Prefecture after high school graduation. It is possible that cases outside Fukushima Prefecture were not captured in the FPCR and were consequently only registered in the FHMS.

In 57.1% of the cases included only in the CR group, thyroid cancer was detected incidentally during follow‐up for another health condition. For > 95% of cases found in both groups, thyroid cancer was detected by screening, including in the FHMS. In the cases found only in the CR group, 28.6% of thyroid cancer cases were identified during screening.

The NCR manual states that “If a patient is suspected of having cancer and visits the hospital, but the examination at that time does not provide any confirmation of cancer, and the patient is followed up for observation and is subsequently diagnosed with cancer, it is considered that a new episode has begun at that time and is considered to have been detected cancer by chance during follow‐up of other diseases.” It is assumed that cases followed up by medical insurance after a detailed examination by the FHMS are included in the group of cases that detected cancer incidentally during follow‐up for another disease. However, the exact number of cases is unknown because some cancer registrars register similar cases as screening findings. Of the 24 cases that were detected incidentally during the follow‐up of another health condition, 22 cases had a history of FHMS examination, and 21 cases were recommended to visit a hospital based on TUE findings, indicating that most cases visited a hospital through the FHMS.

Furthermore, there was no difference in clinical stage, suggesting that most cases were detected by the FHMS and treated after follow‐up under public health insurance. Yokoya et al. [8] have already reported that some cases treated after follow‐up under public health insurance are considered outside of the FHMS cases. Yokoya et al. [8] indicated that, of the 158 cases of thyroid cancer surgically treated at the Fukushima Medical University, 11 were not registered in the FHMS and that seven of these were undergoing treatment after follow‐up under public health insurance.

The increase in the number of cases identified only in the CR group in recent years (Table 2) likely reflects the decrease in participation rates in the FHMS and the increase in cases transferred to public health insurance–based medical care after the TUE program.

The reasons why cases were only registered in the FHMS are as follows. First, it is expected that a certain number of cases in the only FHMS group have not yet been registered in the CR. In the FHMS, all diagnoses are based on fine‐needle aspiration (FNA) cytology results confirming malignancy or potential malignancy, with most diagnosed cases treated surgically. However, until 2015, the date of diagnosis obtained in the FHMS could not be registered as the date of diagnosis in the FPCR. Accordingly, the date of the first hospital visit was used as the date of registration in the FPCR. As a result, the registration date was later in the FPCR than in the FHMS (Table 3). Since 2016, the rules have changed so that if a case was treated at Fukushima Medical University Hospital after the FHMS cytology was performed at Fukushima Medical University Hospital, the date of that cytology can be used as the registration date for the NCR. For cases registered in the FHMS diagnosed using cytology at other hospitals and treated at Fukushima Medical University Hospital, the date of the first visit is the registration date in the NCR as before. The effect of this rule change has been to reduce the number of cases with discrepancies between the registry date of the FHMS and the CRs. There may also be cases of thyroid cancer that have been detected by the FHMS that have not yet been seen in a hospital. Second, since the NCR was established in 2016, the registration of cases only in the FHMS has decreased (Table 2). This indicates that, up to 2015, a certain number of cases registered in only the FHMS group underwent surgical treatment outside of Fukushima Prefecture. The regional analysis revealed that, until 2015 (Figure 3A), more individuals with thyroid cancer registered in only the FHMS group lived in the evacuation area and in Hamadori, located next to the evacuation area, at the time of the disaster, which explains the surgical treatment provided outside of Fukushima Prefecture. In addition, FPCR registration was not mandatory, so even if the surgery had been performed in Fukushima Prefecture, some cases could have been omitted from the registry. Accordingly, the NCR can capture cases more widely than the FPCR.

The present study highlights the importance of the NCR. The NCR needs further development. In addition, in the event of a disaster, a registration system separate from CRs will be required to track migration due to evacuation. Accurate data on the number of cancer cases per region could be used for cancer prevention measures in the event of future accidents. Such information would be necessary in this world where nuclear power plants are still in operation.

The limitations of our study should be acknowledged. First, relocation history based on resident registration was not included. Matching our data with residency cards would provide a history of addresses, which would yield a more accurate identification of individuals. Second, until 2015, only the FPCR data were used for the CRs data for thyroid cancer cases. Considering that many young people moved out of the Fukushima Prefecture after the earthquake, the FPCR registry might not accurately indicate the detection of thyroid cancer. Third, this study is based on information from TUE‐eligible cases. Therefore, cases of thyroid cancer diagnosed without the TUE program and registered in the CRs with an address different from that registered in the FHMS were not included, even if these individuals would have been eligible for the TUE program. Finally, potential biases and confounding factors could not be examined due to the lack of detailed information on each case other than that presented in this study.

Of note, in January 2016, the government of Japan adopted individual numbers as part of the social security and tax number system, with individual number cards used as health insurance cards since March 2021. Centralization of individual numbers and medical information will improve the accuracy of health information within each region.

## 5 Conclusions

To the best of our knowledge, this is the first use of merged data from the CR and FHMS registries to evaluate the detection of thyroid cancer in Fukushima Prefecture. Merging of data from these registries was necessary to more fully capture the thyroid cancer cases after the 2011 Fukushima Daiichi Nuclear Power Plant accident. The NCR, established in 2016, is more precise in capturing cancer cases compared to the local CRs, and it needs further development to become more precise.

## Author Contributions

**Reiko Kimura‐Tsuchiya:** conceptualization (equal), data curation (equal), formal analysis (equal), methodology (equal), validation (equal), visualization (equal), writing – original draft (lead), writing – review and editing (equal). **Masanori Nagao:** conceptualization (equal), data curation (equal), formal analysis (lead), investigation (equal), methodology (equal), resources (equal), software (lead), validation (equal), visualization (lead), writing – original draft (equal), writing – review and editing (equal). **Shigehira Saji:** conceptualization (equal), funding acquisition (equal), methodology (equal), project administration (equal), supervision (equal), writing – review and editing (lead). **Fumikazu Hayashi:** data curation (equal), resources (equal), software (equal), writing – review and editing (equal). **Tetsuya Ohira:** conceptualization (equal), funding acquisition (equal), methodology (equal), project administration (equal), supervision (lead), writing – review and editing (equal). **Hiroki Shimura:** funding acquisition (equal), investigation (equal), resources (equal), supervision (equal), writing – review and editing (equal). **Fumihiko Furuya:** investigation (equal), resources (equal), supervision (equal), writing – review and editing (equal). **Satoru Suzuki:** investigation (equal), resources (equal), supervision (equal), writing – review and editing (equal). **Satoshi Suzuki:** investigation (equal), resources (equal), writing – review and editing (equal). **Tetsuo Ishikawa:** supervision (equal), writing – review and editing (equal). **Susumu Yokoya:** supervision (equal), writing – review and editing (equal). **Hitoshi Ohto:** conceptualization (equal), funding acquisition (equal), project administration (equal), supervision (equal), writing – review and editing (equal). **Seiji Yasumura:** conceptualization (equal), funding acquisition (lead), methodology (equal), project administration (equal), supervision (lead), validation (equal), writing – review and editing (equal).

## Ethics Statement

This study was approved by the Ethics Review Committee of Fukushima Medical University (approval number: 1318, 2020–011) and conducted in accordance with the Helsinki Declaration of the World Medical Association.

## Consent

In the Fukushima Health Management Survey, written informed consent was obtained from the parents or guardians of all survey participants aged < 18 years of age and from the individuals themselves for those aged ≥ 18 years of age.

## Conflicts of Interest

The authors declare no conflicts of interest.

## Permission to Reproduce Material From Other Sources

Access to the National Cancer Registry and Fukushima Prefectural Cancer Registry datasets was obtained with permission from the governments of Japan and the Fukushima Prefecture under the Cancer Registry Promotion Act.

## Data Availability

The data used in this study cannot be shared for privacy or ethical reasons.
